# Effects of a Plant-Focused Diet on the Nutritional Status of Malnourished Patients Undergoing Peritoneal Dialysis in a Selected Hospital Care Setting: Protocol for an Open-Label, Parallel-Group, Randomized Controlled Trial

**DOI:** 10.2196/93558

**Published:** 2026-05-12

**Authors:** Qiao Qian Soon, Harvinder Kaur Gilcharan Singh, Sharmela Sahathevan, Wan Rohaslizan Wan Daud, Muhammad Yusuf Abu Shamsi, Rozita Mohd, Zahara Abdul Manaf

**Affiliations:** 1 Centre for Community Health Studies (ReaCH), Faculty of Health Sciences Universiti Kebangsaan Malaysia Kuala Lumpur, Wilayah Persekutuan Kuala Lumpur Malaysia; 2 Division of Nutrition, Dietetics and Food Science, School of Health Sciences IMU University Kuala Lumpur, Kuala Lumpur Malaysia; 3 Hospital Canselor Tuanku Muhriz, Universiti Kebangsaan Malaysia Cheras, Wilayah Persekutuan Kuala Lumpur Malaysia; 4 Faculty of Medicine Hospital Canselor Tuanku Muhriz, Universiti Kebangsaan Malaysia Cheras, Wilayah Persekutuan Kuala Lumpur Malaysia; 5 Centre for Healthy Ageing and Wellness (H-CARE), Faculty of Health Sciences Universiti Kebangsaan Malaysia Kuala Lumpur, Kuala Lumpur Malaysia

**Keywords:** peritoneal dialysis, malnutrition, plant-based diet, plant-focused diet, nutritional status, medical nutrition therapy, randomized controlled trial, renal nutrition, Malaysia

## Abstract

**Background:**

Malnutrition is a common and serious complication among patients undergoing peritoneal dialysis (PD), leading to poor clinical outcomes, reduced quality of life, and increased mortality. Although current renal dietary guidelines emphasize adequate protein intake, they are predominantly animal based and may exacerbate inflammation and metabolic complications. Emerging evidence suggests that plant-focused diets can improve nutritional and inflammatory profiles without raising serum potassium levels. Increased plant consumption has also been associated with better metabolic control, reduced inflammation, and improved bowel function in patients undergoing PD. However, randomized controlled trials remain limited, and the effectiveness of such diets in malnourished patients undergoing PD is unclear. Hence, further investigation is required to evaluate the efficacy and safety of a plant-focused diet to inform evidence-based dietary recommendations in this population.

**Objective:**

This study aims to determine the effectiveness of a plant-focused dietary intervention compared with a standard-of-care renal diet on nutritional status, measured by changes in serum albumin, among malnourished patients undergoing PD over 6 months.

**Methods:**

This is an unblinded, open-label, parallel-group randomized controlled trial conducted at a tertiary care hospital in Klang. A total of 100 adult outpatients undergoing PD will be recruited and randomized (1:1) to receive either a plant-focused diet emphasizing plant-based proteins or a standard renal diet emphasizing animal-based proteins for 6 months. Both interventions will be individualized to meet nutrient requirements and delivered through dietitian-led counseling, educational materials, and regular follow-ups. Data will be collected at baseline and after 3 and 6 months of intervention. Parameters assessed include sociodemographic characteristics; anthropometric and biochemical measures (renal, glucose, lipid, and inflammatory profiles); malnutrition inflammation score; dietary adequacy; physical activity and function; quality of life; and knowledge, attitude, and practices of renal diet. Baseline data will be analyzed using descriptive statistics, with independent *t* tests (2-tailed) or Mann-Whitney *U* tests for between-group comparisons. Changes across time points will be analyzed using a generalized linear model for repeated measures, with Bonferroni adjustment for multiple comparisons. Analyses will be adjusted for confounders with significance set at *P*<.05. Ethics approval has been obtained from the Universiti Kebangsaan Malaysia Research Ethics Committee (JEP-2025-812).

**Results:**

This study was funded in 2024 under the *Geran Galakan Penyelidik Muda* (project code: GGPM-2024-051) by Universiti Kebangsaan Malaysia. Participant recruitment began in March 2026, and 9 participants have been recruited as of manuscript submission. Study is expected to be completed by March 2027 Data analysis and manuscript preparation are anticipated to be completed by June 2027.

**Conclusions:**

This randomized controlled trial will provide clinical evidence on the nutritional and safety outcomes of a plant-focused diet in malnourished patients undergoing PD, addressing a major evidence gap in renal nutrition management.

**Trial Registration:**

ClinicalTrials.gov NCT07157397; https://clinicaltrials.gov/study/NCT07157397 and National Medical Research Register NMRR ID-26-00114-LAK; https://nmrr.gov.my/research-directory/d0aad3f4-46ac-4498-8b75-f82f02209cec

**International Registered Report Identifier (IRRID):**

PRR1-10.2196/93558

## Introduction

### Background

According to the Kidney Disease: Improving Global Outcomes guidelines, end-stage renal disease (ESRD) is defined as irreversible kidney failure persisting for more than 3 months [1]. The prevalence of ESRD has significantly increased over the years alongside the growing rates of diabetes and hypertension, both of which are key risk factors for the development and progression of chronic kidney disease (CKD) [2]. ESRD represents the final stage of CKD, in which kidney functions are severely impaired and no longer sufficient to sustain life. As such, renal replacement therapy (RRT) becomes necessary. RRT options include hemodialysis, peritoneal dialysis (PD), and kidney transplantation. However, in Malaysia, due to limitations for organ transplant, dialysis remains the most commonly used mode of RRT [3]. The number of patients requiring RRT in Malaysia has notably increased, with those undergoing PD rising from 13% in 2014 to 18% in 2023, reflecting the growing role of PD in RRT [3].

Despite its benefits, PD is associated with various complications, particularly malnutrition and protein-energy wasting. A systematic review and meta-analysis reported that patients undergoing PD have the highest global prevalence of malnutrition at 45.3%, compared to 43.1% in patients undergoing hemodialysis and 38.5% in nondialysis individuals [4]. The 31st Report of the Malaysian Dialysis and Transplant Registry indicated a widespread malnutrition among patients undergoing PD in Malaysia, with only 27% of patients undergoing PD in half the centers meeting both the recommended body mass index (BMI) and serum albumin targets in 2023 [3]. Additionally, 48% were overweight or obese (BMI≥25 kg/m²), while 8% were underweight (BMI<18.5 kg/m²), highlighting the coexistence of overnutrition and undernutrition [3]. A study conducted among the dialysis population in Malaysia also found a high prevalence of malnutrition among patients undergoing PD, with >70% classified as malnourished across 3 different assessment criteria [5]. Macronutrient and micronutrient deficiencies in patients undergoing PD can result from factors such as inadequate dietary intake, impaired intestinal function, losses through dialysate and urine, metabolic imbalances, chronic inflammation, and the hypercatabolic state of ESRD [6-8]. Poor oral intake is a major contributor to malnutrition among this population. A study conducted in Malaysia reported that the average energy intake among patients undergoing PD ranged from 23 to 25 kcal/kg/day, with protein intake at approximately 0.82 g/kg/day, which are both below recommended levels [9]. Studies have shown that malnutrition in patients undergoing PD result in lower quality of life (QOL) and higher risks of morbidity and mortality [10-12]. Given the strong association between poor nutrition and adverse health outcomes, effective dietary strategies are essential. Therefore, further research is essential to improve nutritional strategies for disease management, ultimately improving patient outcomes and QOL.

Recent evidence suggests that plant-based diets may offer benefits for patients undergoing dialysis by reducing blood pressure, metabolic acidosis, and gut-derived uremic toxins without significantly increasing serum potassium levels [13,14]. Nonetheless, concerns remain regarding the safety of these diets in this population, including risks of hyperkalemia, hyperphosphatemia, and inadequate protein or energy intake [15]. Moreover, most existing evidence on plant-focused or plant-based diets is derived from the broader CKD population, with limited data specific to patients undergoing PD. Although observational studies suggest potential advantages, clinical trials investigating the effects of plant-focused diets in patients undergoing PD are lacking, highlighting a significant gap in the current literature that this study aims to address [1]. Additionally, no studies to date have directly compared plant-focused diets with standard renal dietary recommendations in this population. Hence, findings from this study may offer valuable insights to inform future dietary guidelines for PD populations.

### Research Problem

Malnutrition is highly prevalent among patients undergoing PD [3-5]. Although plant-focused diets have shown potential benefits in reducing mortality, uremia, metabolic acidosis, and inflammation, their safety and effectiveness in patients undergoing PD remain unclear [15]. Limited clinical trial evidence highlights the need for further research to evaluate their impact on nutritional outcomes and overall safety in the PD population [1].

### Research Question

The study is guided by the following research questions:

- Does a plant-focused dietary intervention improve nutritional status, measured by changes in serum albumin, compared with a standard renal diet among malnourished patients undergoing PD over 6 months?
- What is the effect of plant-focused diet in comparison to standard-of-care renal diet on other nutritional outcomes such as anthropometric measurements (BMI, triceps skinfold [TSF], and midarm muscle circumference [MAMC]), biochemical and inflammatory marker (lipid profile, high-sensitivity C-reactive protein [hs-CRP], and renal parameters), dietary intake, malnutrition inflammation score (MIS), assessment of physical function, QOL, appetite, and retention rate in the trial among malnourished patients undergoing PD over 6 months?

### Research Hypotheses

The first alternative hypothesis is as follows: the serum albumin level improved significantly among malnourished patients undergoing PD following a plant-focused diet compared to a standard-of-care renal diet after 6 months of intervention.

The second alternative hypothesis is that other nutritional outcomes, such as anthropometric measurements (BMI, TSF, and MAMC), biochemical and inflammatory marker (lipid profile, hs-CRP, and renal parameters), dietary intake, MIS scores, assessment of physical function, QOL, appetite, and retention rate in the trial, improved significantly among malnourished patients undergoing PD following the plant-focused diet in comparison to a standard-of-care renal diet after 6 months of intervention.

### Research Objectives

The primary objective is to determine the effect of a plant-focused dietary intervention on changes in serum albumin from baseline to 6 months compared with standard-of-care renal diet in malnourished patients undergoing PD.

The secondary objectives are (1) to determine the effect of a plant-focused dietary intervention on changes in anthropometric measurements (BMI, TSF, and MAMC), biochemical and inflammatory markers (lipid profile, hs-CRP, and renal parameters), dietary adequacy and nutrient intake, MIS scores, physical function and muscle strength, QOL, appetite scores, and dialysis adequacy from baseline to 6 months compared with standard-of-care renal diet in malnourished patients undergoing PD and (2) to determine the baseline nutritional status of malnourished patients undergoing PD, including their knowledge, attitude, and practices of renal diet intake, as well as sociodemographic characteristics and medical history.

Exploratory objectives are to assess adherence to a plant-focused dietary intervention over the 6-month period and to evaluate retention rates over 6 months of intervention.

## Methods

### Study Design

#### Overall Study Design

This study is an unblinded, open-label, parallel-group randomized controlled trial evaluating the effect of a plant-focused diet on the nutritional status of malnourished patients undergoing PD. This study will be conducted over 6 months, alongside each participants’ routine PD clinic visits. Patients and the public were not involved in the design, conduct, reporting, or dissemination plans of this study.

#### Research Flow

**Figure 1:**
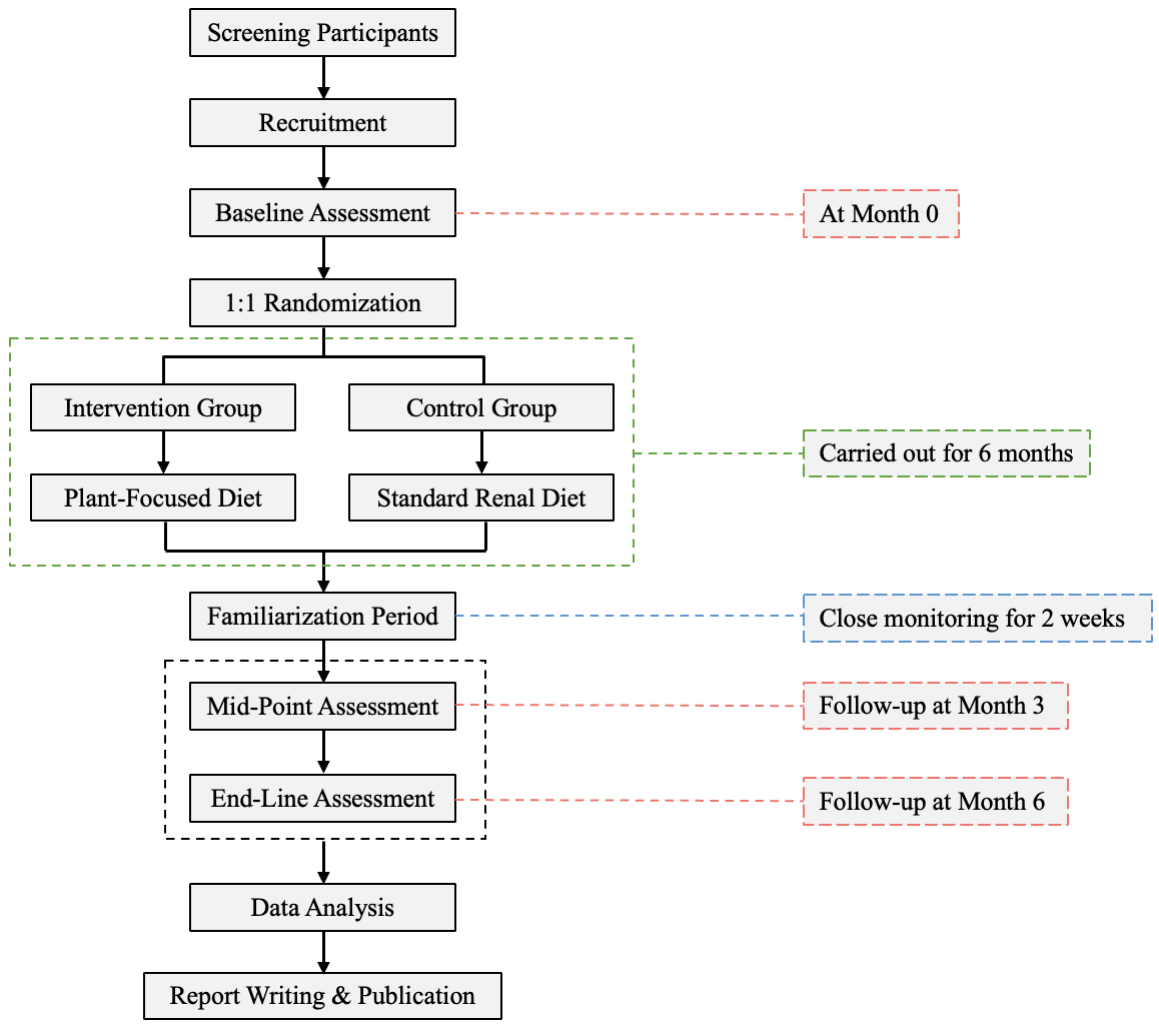
Research flowchart.

This study protocol is adapted from a similar ongoing clinical trial involving patients with CKD with diabetes mellitus in the United States [16]. Figure 1 shows a diagram outlining the research flow. The study begins with participant screening, recruitment, and baseline assessment conducted on the same day. Following baseline assessment, participants are then randomized into either the intervention group receiving a plant-focused dietary intervention or the control group receiving a standard renal dietary intervention.

A 2-week familiarization phase is implemented immediately after randomization as part of the intervention period. During this phase, participants begin following their assigned dietary intervention and receive additional guidance and closer monitoring to support early adaptation and adherence. This phase is intended to facilitate understanding of the dietary protocol and to identify and address potential challenges early, rather than to assess eligibility or introduce selection bias.

Participants from both groups are instructed to continue the assigned dietary intervention for 6 months. Key outcome assessments are conducted at baseline (0 months), midpoint (3 months), and end line (6 months). Data collected will be analyzed for subsequent report writing and publication.

The intervention and study procedures, including screening, recruitment, data collection, and intervention delivery, will be conducted by a registered dietitian. Health care staff at the study site will assist with clinical procedures such as blood collection.

### Recruitment

#### Study Population

Participants will be recruited from the nephrology unit at a tertiary care hospital in Klang, following approval from the research ethics committee. A second center may be considered to meet recruitment targets.

#### Sample Size

The sample size was calculated using the standard formula for comparing 2 independent means in a parallel-group design using several assumptions.

On the basis of previous studies in dialysis populations, an estimated SD of 5 g/L was used to account for heterogeneity and to avoid underestimation of sample size. Notably, studies by Kalantar-Zadeh et al [17] and Rambod et al [18] reported serum albumin SDs ranging from 1.9 to 4.4 g/L, while a study by Limwannata et al [19] reported an SD of 5.0 g/L.

A clinically meaningful difference (δ) of 3 g/L (0.3 g/dL) was selected as it is consistent with differences in serum albumin levels observed across dialysis populations in previous studies. For instance, variations of 0.3 to 0.4 g/dL are observed between cohorts reported in studies by Rambod et al [18] and Limwannata et al [19]. Additionally, serum albumin is a well-established predictor of morbidity and mortality in patients undergoing dialysis, and even modest changes are considered clinically relevant in reflecting improvements in nutritional status [17].

Using the formula mentioned below, a 2-sided significance level of.05 (Zα/2=1.96) and 80% power (Zβ=0.84) were used to calculate the sample size. As a result, a minimum of 44 participants per group was required. After accounting for an anticipated 10% dropout rate, the sample size was rounded up to 50 participants for each group, with a total sample size of 100 participants. As repeated measurements will be conducted at baseline, 3 months, and 6 months, the calculated sample size is considered conservative.

The sample size was calculated using the standard formula for comparing 2 independent means in a parallel-group design, as shown below:



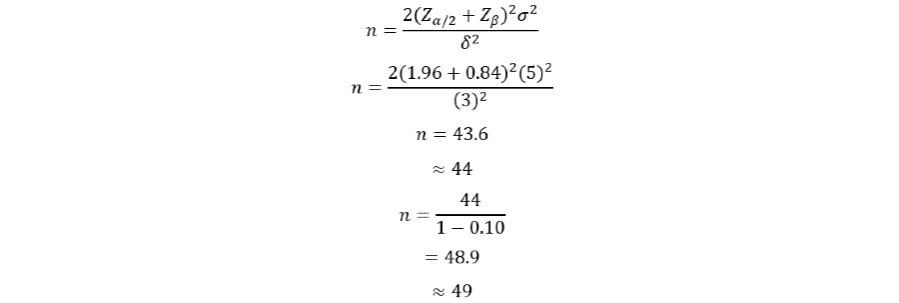



Where n is the sample size per group, Zα*/*2 is the standard normal deviate for a 2-sided significance level (1.96 for *α*=.05), Zβ is the standard normal deviate for power (0.84 for 80% power), σ is the SD, and δ is the expected difference between groups.

#### Participant Screening and Recruitment

Screening of participants will be conducted by reviewing the patient registry at the identified PD clinic to identify potential participants. Medical records will then be used to assess eligibility, and those who do not meet the criteria, as shown in Textbox 1, will be excluded. Eligible participants will be contacted via phone to receive a brief explanation of the study, and those who have expressed interest in participating or provided verbal consent will be scheduled for a physical screening visit at the clinic. During the screening visit, the participants will be provided with the participant information sheet and informed consent form, available in both Malay and English languages. They will be given ample time to review the study details before providing written consent, and only participants who have signed the consent form will be assigned a screening ID.

**Participant inclusion and exclusion criteria.**

**Inclusion criteria**

- Adult outpatients (aged >18 years) attending peritoneal dialysis (PD) clinic visits
- Have undergone peritoneal dialysis for at least 6 months
- Have a serum albumin level <40 g/L
- Have a malnutrition inflammation score of mild, moderate, or severe
- Agreed to follow the dietary instructions based on the randomization assignment
- Agreed to attend baseline visits and additional follow-up appointments at months 1, 3, and 6 postrandomization, either in person or via telehealth, and respond to monthly or more frequent phone calls

**Exclusion criteria**

- Have been hospitalized for the past 3 months
- Have ongoing infection or sepsis
- Has undergone surgery in the past 6 months
- Have high inflammatory diseases, malignancy, or cancer
- Have dialysis adequacy of <1.2 kt/V

#### Participant Withdrawal and Dropouts

Throughout the 6-month study period, the participants will be monitored for adherence and motivation. The specific criteria for withdrawal and dropout are as follows: (1) missing ≥50% of follow-up visits during the 6-month study, (2) <50% compliance at 3 consecutive monthly review points based on the dietary compliance scoring system (scoring criteria are informed by clinical relevance, current dietary guidelines, and prior literature), (3) failure to comply with the study protocol, (4) voluntary withdrawal from the study, and (5) identification of a clinical condition requiring intervention or likely to affect study outcomes.

To manage dropouts, participants who withdraw, fail to meet compliance, or are lost to follow-up will be seen by an investigator. Reasons for failure to comply with the study protocol will be documented. Data collected up to the point of withdrawal will be retained and analyzed unless requested otherwise by the participant. Participants withdrawn from the study will not be replaced, as the sample size calculation includes an allowance for dropouts.

### Randomization

#### Randomization Procedures

Participants will be randomly assigned using the Random Allocation Software (version 2.0; developed by Mahmood Saghaei, Isfahan University of Medical Sciences) to either the intervention group (plant-focused dietary intervention) or the control group (standard renal dietary intervention) [20]. The software will generate allocation codes (1 for intervention and 2 for control) based on block randomization with a 1:1 ratio and a block size that is a multiple of 4. This study is unblinded, and the allocation sequence will be accessible to the researcher assigning participants.

#### Intervention Group vs Control Group

The control group will follow a standard renal diet and be encouraged to maintain their usual dietary and physical activity patterns, with guidance to ensure that at least 50% of their total protein intake comes from high biological value animal-based sources. The standard renal diet was chosen as comparator as it reflects the current clinical practice. In contrast, the intervention group will follow a plant-focused dietary pattern that emphasizes plant-based foods, such as fruits, vegetables, legumes, whole grains, nuts, seeds, and plant oils, with minimal reliance on animal-based products. Participants in the intervention group will be required to consume plant-focused meals for most of their daily meals. For example, those consuming 3 meals per day must ensure that at least 2 out of the 3 meals are plant based, while those consuming 4 meals per day must ensure that at least 3 out of the 4 meals are plant based. The remaining meal or snack may include either plant- or animal-based foods to support flexibility and adherence.

Participants in both groups will receive individualized dietary counseling at each data collection point (baseline, 3 months, and 6 months). In addition, participants are also provided with educational pamphlets and 14-day structured meal plans that incorporate natural food sources and culturally appropriate meal choices. Meal plans for both groups are developed in accordance with the Malaysian Medical Nutrition Therapy Guidelines for Chronic Kidney Disease and the Kidney Disease Outcomes Quality Initiative Clinical Practice Guidelines for Nutrition in CKD [7,21]. Throughout the study, participants will continue to receive standard medical care, with no specific concomitant care prohibited.

### Data Collection Procedures

#### Overview

The parameters assessed at each participant visit, from screening through the 6-month study duration, are summarized in Table 1. Data will be collected through various questionnaires and standardized procedures. All questionnaires used are freely and publicly accessible.

**Table 1:** Summary of parameters assessed during participant visits.

Variables			Screening		Month 0		Month 3		Month 6
**Anthropometric data**									
	Weight	✓		✓		✓		✓	
	Height	✓		✓		✓		✓	
	BMI	✓		✓		✓		✓	
	Triceps skinfold	✓		✓		✓		✓	
	MAMC^a^	✓		✓		✓		✓	
	Body composition	✓		✓		✓		✓	
**Biochemical data**									
	Renal function test	✓		✓		✓		✓	
	Lipid profile	✓		✓		✓		✓	
	Blood glucose profile	✓		✓		✓		✓	
	Dialysis adequacy (kt/V)	✓		✓		✓		✓	
	nPCR^b^	✓		✓		✓		✓	
	Inflammatory markers			✓		✓		✓	
**Clinical data**									
	MIS^c^ score	✓		✓		✓		✓	
**Dietary assessment**									
	Food records	✓		✓		✓		✓	
	Appetite and food satisfaction			✓		✓		✓	
**Physical activity and function**									
	SPPB^d^			✓		✓		✓	
	Handgrip strength			✓		✓		✓	
	Physical activity record	✓		✓		✓		✓	
**Others**									
	Sociodemographic data and medical history	✓		✓					
	Knowledge, attitude, and practices	✓		✓					
	Medication log	✓		✓		✓		✓	
	Quality of life (WHOQOL-BREF^e^)			✓		✓		✓	
	Adverse events report					✓		✓	

^a^MAMC: midarm muscle circumference.

^b^nPCR: normalized protein catabolic ratio.

^c^MIS: malnutrition inflammation score.

^d^SPPB: Short Physical Performance Battery.

^e^WHOQOL-BREF: World Health Organization Quality of Life-BREF.

#### Anthropometric Data

Postdialysis weight, height, BMI, TSF, and mid–upper arm circumference will be measured using standard protocols. MAMC will be calculated as *MAMC=mid*–*upper arm circumference−(π×TSF)*. Body composition will be assessed using bioelectrical impedance analysis where available.

#### Biochemical Data

Laboratory parameters (renal, lipid, glucose, inflammation, dialysis adequacy, and protein intake) will be extracted from hospital records. hs-CRP will be analyzed externally using 10 mL fasting blood samples collected by trained staff.

#### Malnutrition Inflammation Score

Nutritional and inflammatory status will be assessed using the MIS (range 0-30). This tool has been locally validated, with a score of <5 indicating normal status and ≥5 indicating malnutrition [5].

#### Dietary Assessment and Compliance Toward Diet Regime

Participants will complete a 3-day food diary monthly (2 weekdays and 1 weekend). At the first clinic visit, participants will receive training on how to accurately record only foods and beverages consumed using household measurements and a colored food album to assist with portion size estimation. Dietary intake data will then be analyzed using Nutritionist Pro (version 5.1.0; Axxya Systems [22]) using information from the Nutrient Composition of Malaysian Foods and Singapore Food Composition Book (Ministry of Health Singapore [23,24]).

Dietary adherence will be evaluated using a binary compliance scoring system, as detailed in Tables 2 and 3. Although arbitrarily set, the scoring categories were informed by clinical relevance, current dietary guidelines (medical nutrition therapy and Kidney Disease Outcomes Quality Initiative), and prior literature on compliance assessment in chronic disease populations [7,21,25].

**Table 2:** Dietary compliance scoring system (scored per day).

Category				Scoring criteria^a^		
				Score=0		Score=1
**Meal composition**						
	Meal frequency		Skipped ≥1 main meal/day		Consumed 3-4 main meals/day, without skipping	
	**Protein source**					
		Control	<50% protein intake from HBV^b^ animal-based sources		≥50% protein intake from HBV animal-based sources	
		Intervention	<2/3 or 3/4 meals are plant-based food sources		≥2/3 or 3/4 meals are plant-based food sources	
**Nutrient intake**						
	Total energy intake		Deviation of >+20% or −20% of target energy prescription		Within +20% or −20% of target energy prescription	
	Total protein intake		Deviation of >+20% or −20% of target protein prescription		Within +20% or −20% of target protein prescription	
	Total sodium intake		Outside 2000-3000 mg/day		Within 2000-3000 mg/day	
	Total potassium intake		Outside 2000-3000 mg/day		Within 2000-3000 mg/day	
	Total phosphate intake		Outside 0.8-1.0 g/day		Within 0.8-1.0 g/day	

^a^Total scores=0-7 (each daily dietary record is scored out of 7 points).

^b^HBV: high biological value.

**Table 3:** Diet compliance score classification.

	Per day (1-day)	Per monthly review (3-day)	Per monthly review (3-day)
Maximum score	7	21	21
Total score	—^a^	<10.5/21 (<50%)	≥10.5/21 (≥50%)
Classification	—	Poor compliance	Adequate compliance

^a^Not applicable.

Each daily dietary record is scored out of a maximum of 7 points. A “1-day” record refers to a single 24-hour dietary record, whereas a “3-day” record represents the aggregation of three separate daily dietary records collected from participants. The total score for each 3-day dietary assessment therefore ranges from 0 to 21 points. Total scores are calculated by summing the scores from each daily record. A total score of ≥50% (≥10.5/21) indicates adequate dietary compliance, whereas scores <50% indicate poor compliance. To ensure feasibility and acceptability, the dietary compliance scoring system will be pilot tested with participants during the first 2 weeks of the study period.

#### Appetite and Food Satisfaction Assessment

Appetite will be assessed using the validated Appetite and Food Satisfaction Questionnaire, which scores appetite and satisfaction from 0 to 18 (lower=better appetite) [26].

#### Quality of Life

QOL will be measured using the World Health Organization Quality of Life-BREF, comprising 26 items across 4 domains (physical, psychological, social, and environmental) [27]. Scores are converted to a 0 to 100 scale, with higher scores reflecting better QOL.

#### Physical Activity and Function

Physical activity will be measured using the short form of the International Physical Activity Questionnaire [28]. Results will be expressed in Metabolic Equivalent of Task minutes per week. Physical function will be assessed using the Short Physical Performance Battery, a well-established, standardized instrument that has been extensively used and validated in dialysis populations worldwide, with higher total scores indicating better physical function [29,30]. Handgrip strength will be measured using a Jamar dynamometer, with the mean of 3 consistent trials recorded.

#### Knowledge, Attitude, and Practices on Renal Diet

A validated 34-item questionnaire will assess dietary knowledge (12 items), attitude (7 items), and practice (15 items) [31]. Higher scores reflect better understanding and adherence to renal diet principles.

#### Sociodemographic and Medical History

Sociodemographic and medical history are collected via a structured interview guided by a general questionnaire covering demographic data, socioeconomic status, medical and dialysis history, medications, physical activity, and allergies.

### Statistical Analysis

Statistical methods are detailed in Table 4. Data will be analyzed using SPSS (version 28; IBM Corp). Normality of continuous data will be tested using the Kolmogorov-Smirnov test.

**Table 4:** Summary of the statistical analysis.

Study outcomes	Variables	Statistical analysis
Primary or main outcome	Serum albumin	GLM^a^-repeated measures (time×group interaction)
Secondary outcomes	Anthropometric data Biochemical data Dietary adequacy Physical function Quality of life Appetite and food satisfaction Dialysis adequacy	GLM-repeated measures (time×group interaction)
Exploratory or feasibility outcomes	Dietary adherence or compliance Retention rate	Descriptive statistics and chi-square test

^a^GLM: generalized linear model.

The primary analysis will evaluate changes in serum albumin from baseline to 3 and 6 months between intervention and control groups. A generalized linear model for repeated measures will be used to assess time, group, and interaction effects. Analyses will follow the intention-to-treat principle, with missing data handled using the last observation carried forward method. Adjustment for potential confounders such as age, sex, dialysis duration, and baseline nutritional status will be performed using multivariable models. Bonferroni correction will be applied for multiple comparisons. Secondary outcomes, including anthropometric, biochemical, dietary, MIS, physical function, QOL, appetite, and dialysis adequacy parameters, will be analyzed using similar repeated-measures approaches. Statistical significance will be set at *P*<.05.

### Trial Registration

This study has been registered with ClinicalTrials.gov (NCT07157397) and National Medical Research Register (NMRR ID-26-00114-LAK).

### Ethical Considerations

#### Ethics Approval

This study has obtained ethics approval from the Universiti Kebangsaan Malaysia Research Ethics Committee (JEPUKM; JEP-2025-812). The study will be conducted in accordance with the Declaration of Helsinki, Malaysian Good Clinical Practice Guidelines, and institutional policies. Any protocol deviations will be reported to the ethics committee as required. Adverse events will be monitored throughout the study period and followed up as appropriate. Participants will continue to receive standard clinical care throughout the trial.

A data monitoring committee is not required for this study due to the low-risk nature of the intervention. No interim analyses or stopping guidelines are planned due to the nature and duration of the study. Trial conduct will be monitored by the research team to ensure adherence to the study protocol. No formal external monitoring is planned due to the low-risk nature of the study.

#### Informed Consent

Eligible participants will receive a participant information sheet and informed consent forms, available in Malay and English. Written consent will be obtained before any study-related procedures. If written consent is not possible, oral consent will be documented in the presence of an independent witness. Participation is voluntary, and participants may withdraw at any time without affecting their standard care. Data collected up to withdrawal will be retained unless otherwise requested.

#### Data Confidentiality and Protection

All participant data will be anonymized and handled in compliance with the Malaysian Personal Data Protection Act 2010 [32]. Data will be stored on drives accessible only to authorized personnel and retained for at least 2 years after study completion before secure disposal. Results will be disseminated through peer-reviewed publications, conference presentations, and individual summaries shared with participants where appropriate. No personal identifiers will appear in any publications. Deidentified data will not be publicly available due to ethical and privacy considerations.

## Results

### Study Timeline

Participant recruitment began in March 2026, and the study is expected to conclude by March 2027. As of manuscript submission, 9 participants have been recruited. Data analysis and manuscript writing are anticipated to be completed by June 2027.

### Planned Outputs

The research team plans to publish three manuscripts from this project: (1) this study protocol; (2) a secondary cross-sectional analysis using baseline data from enrolled participants to examine the association between dietary knowledge, attitudes, and practices and nutritional outcomes among patients undergoing PD; and (3) the final findings on the effectiveness of the intervention after study completion.

## Discussion

### Significance of the Study

This paper outlines the protocol for a randomized controlled trial designed to evaluate the effects of a plant-focused diet on nutritional and clinical outcomes among malnourished patients undergoing PD in a selected hospital setting in Malaysia. This research is novel and has not been conducted in the ESRD or PD population. Currently, only 1 ongoing study in the United States is examining the effects of plant-focused diets in patients with CKD [16].

### Purpose of the Study

Over the years, the use of PD has steadily increased in Malaysia by 5% from 13% in 2014 to 18% in 2023, alongside a persistently high prevalence of malnutrition in this population [3]. While nutritional issues in patients undergoing PD can arise from multiple factors, they are largely influenced by inadequate oral intake. As evidenced by a local study, Malaysian patients undergoing PD were reported to only consume an average energy intake of around 23 to 25 kcal/kg/day and 0.82 g protein/kg/day, both of which are below the recommended levels [9]. This poor intake has been attributed to appetite loss, altered taste perception, and fatigue often associated with uremia, metabolic acidosis, and abdominal discomfort or constipation [33].

Moreover, studies have highlighted that patients undergoing PD often lack nutrition knowledge and confidence, leading to fear of eating and self-imposed dietary overrestrictions that further exacerbate their nutritional status [34]. When combined with increased nutritional demands due to inflammation and unintentional nutrient losses during dialysis, these factors significantly compromise the nutritional status of patients undergoing PD, contributing to the development of nutrition-related complications [35]. Addressing nutrition-related complications in this population is essential, as malnutrition has been consistently linked to adverse outcomes, including increased hospitalization, reduced QOL, and higher mortality rates [36].

### Literature Gap and Potential Benefits of the Study

While existing studies have focused on the potential benefits of plant-based or plant-focused diets on the broader CKD population, evidence supporting these diets in ESRD populations remains limited, particularly in the PD population. Additionally, concerns remain regarding potential risks, as there is a notable lack of clinical trials, making it difficult to determine the true safety and efficacy of plant-focused or plant-based diets in PD populations [1,15]. This leaves a significant gap in understanding the safety and efficacy of these diets in patients undergoing PD.

This study seeks to address this literature gap by comparing plant-focused dietary approaches with current renal dietary recommendations. Findings of this study will provide clinically relevant evidence on the safety and effectiveness of plant-focused diets in patients undergoing PD and potentially offer insights to guide future dietary recommendations for patients undergoing PD. In addition, publications from this study may potentially encourage better nutrition practices through better nutrition education. Ultimately, this study has the potential to enhance patient-centered care, improve clinical outcomes, and inform policy-level strategies for the nutritional management of ESRD and PD.

## Appendix

Multimedia Appendix 1 SPIRIT v2025 Checklist.

Multimedia Appendix 2 Peer-review report from Geran Galakan Penyelidik Muda (GGPM) grant committee, Univerisiti Kebangansaan Malaysia.

## Abbreviations

CKD: chronic kidney disease
ESRD: end-stage renal disease
hs-CRP: high-sensitivity C-reactive protein
MAMC: midarm muscle circumference
MIS: malnutrition inflammation score
PD: peritoneal dialysis
QOL: quality of life
RRT: renal replacement therapy
TSF: triceps skinfold
