# Supplementary material for: Effects of a Plant-Focused Diet on the Nutritional Status of Malnourished Patients Undergoing Peritoneal Dialysis in a Selected Hospital Care Setting: Protocol for an Open-Label, Parallel-Group, Randomized Controlled Trial
Source: JMIR Res Protoc. 2026 May 12;15:e93558. doi: 10.2196/93558 (PMC13213326; doi:10.2196/93558)
Supplement: Multimedia Appendix 2 [file resprot_v15i1e93558_app2.pdf]

Reference: Project Code GGPM-2024-051
Date: 16 February 2026

Editor-in-Chief
Journal of Medical Internet Research (JMIR) Research Protocols
Journal of Medical Internet Research (JMIR) Publications Inc.

Dear Editor,

"EFFECT OF PLANT-FOCUSED DIET ON NUTRITIONAL STATUS OF MALNOURISHED PATIENTS UNDERGOING PERITONEAL DIALYSIS IN A SELECTED HOSPITAL CARE SETTING: STUDY PROTOCOL FOR AN OPEN-LABEL, PARALLEL-GROUP, RANDOMISED CONTROLLED TRIAL."

We are pleased to submit the above-mentioned manuscript for consideration for publication in JMIR Research Protocols.

This manuscript presents the protocol of an open-label, parallel-group randomized controlled trial evaluating the effectiveness and safety of a plant-focused dietary intervention compared with standard renal dietary care among malnourished patients undergoing peritoneal dialysis in a tertiary hospital setting in Malaysia. The study addresses an important evidence gap regarding plant-focused dietary strategies in this high-risk population.

The trial has received ethical approval from the Research Ethics Committee, The National University of Malaysia (JEP-2025-812) and is registered at ClinicalTrials.gov (NCT07157397) and the National Medical Research Register (NMRR ID-26-00114-LAK). Recruitment is scheduled to commence in March 2026.

This protocol is submitted prior to participant recruitment to ensure methodological transparency and reproducibility. The manuscript has not been published previously and is not under consideration elsewhere. All authors have reviewed and approved the submission.

We have attached two peer-reviewed reports below for your reference.

We appreciate your time and consideration and look forward to your response.

Thank you.

Yours sincerely,
Soon Qiao Qian
Dietitian, Graduate Research Assistant
Center for Community Health Studies (Reach)
Faculty of Health Sciences
The National University of Malaysia
(On behalf of all co-authors)

EVALUATION OF POSTGRADUATE RESEARCH PROPOSAL
FACULTY OF HEALTH SCIENCES

**Name of Student**	:	**Soon Qiao Qian**
**Matrix No.**	:	**P156337**
**Programme**	:	
**Specialization**	:	
**Supervisory committee**	:	**Dr Harvinder Kaur A/P Gilcharan Singh**
**Research centre**	:	**Reach**

Item	Evaluation Criteria	Rating Score	Comments
**1.**	**Suitability of Research Title**<br><br>(Research title able to demonstrate the meaning and cover the scope of study)	5	Suitable title
**2.**	**Introduction and Literature Review**<br><br>(Suitability of Background information, problem statements and relevant literatures)	5	Relevant literature review.

Item	Evaluation Criteria	Rating Score	Comments
**3.**	**Scientific Merit of Research Justification and Objectives**<br><br>(Research aims/objectives and justifications are well-defined and highlight the scientific merit of the study)	4	- It is unclear how the researcher intends to measure Objective 1.<br>- The stated hypothesis appears to be a null hypothesis, which suggests no significant difference between the intervention and control groups. Could you clarify if this is the intended hypothesis?
**4.**	**Research Methodology**<br><br>(Suitability of research design, structure, approach and planning of research project)	4	- Describe the management of drop out or missing data<br>- Suggest to consider the readiness of change/patient motivation level as one of criteria to screen for participant
**5.**	**Research Impact**<br><br>(Benefits and contributions of the research to the respective field)	5	The study addresses a timely and relevant topic, considering plant-focused dietary interventions, an area with limited prior exploration in the ESRD-PD population

Item	Evaluation Criteria	Rating Score	Comments
6.	**Overall Performance**<br><br>(Presentation skills, visual aids, shows confidence during Q&A session, etc.)	5	Student show a good understanding of research project.
7	**Suitability of the specialization**<br>(if the study is within the scope of specialization.)	-	-
	**Total Score**	28	

Rating Score: 1 = Very Poor, 2 = Poor, 3 = Average, 4 = Good, 5 = Excellent

Decision: **Approved (/)** **Approved with corrections ( )** **Re-write ( )**
Re-presentation ( ) **Rejected ( )**

Signature :

Name of reviewer : Fatin Hanani Mazri

Date : 28 July 2025

EVALUATION OF POSTGRADUATE RESEARCH PROPOSAL
FACULTY OF HEALTH SCIENCES

**Name of Student**	**:**	**Soon Qiao Qian**
**Matrix No.**	**:**	**P156337**
**Programme**	**:**	
**Specialization**	**:**	
**Supervisory committee**	**:**	**Dr. Harvinder Kaur A/P Gilcharan Singh**
**Research centre**	**:**	**Pusat Kajian Kesihatan Komuniti (ReaCH)**

Item	Evaluation Criteria	Rating Score	Comments
**1.**	**Suitability of Research Title**<br><br>(Research title able to demonstrate the meaning and cover the scope of study)	4	-Suggestion to add the duration of study in the title (the intervention period)
**2.**	**Introduction and Literature Review**<br><br>(Suitability of Background information, problem statements and relevant literatures)	4	-Please add references to the statement mentioned<br>-Please follow the UKM style of formatting

Item	Evaluation Criteria	Rating Score	Comments
**3.**	**Scientific Merit of Research Justification and Objectives**<br><br>(Research aims/objectives and justifications are well-defined and highlight the scientific merit of the study)	4	-Include the term “intervention” in the main objective<br>-Please revise the specific objectives, the statement should be the outcome measures, and not mention secondary or primary<br>-Please revise the way of writing a hypothesis<br>-Add more input on the study's significance and justification
**4.**	**Research Methodology**<br><br>(Suitability of research design, structure, approach and planning of research project)	4	-Add a detail on the ethical application from JEPUKM. If this study has received ethical approval, then please state the number/code.<br>-What is the reference for Table 3.3? Do you adapt the prescription from a previous study? Please add information about the prescription<br>-What is the reference for information in Table 3.4?<br>-Please describe the statistical analysis according to each specific objective. You can also summarize in a table
**5.**	**Research Impact**<br><br>(Benefits and contributions of the research to the respective field)	3	-Please add this in the proposal

Item	Evaluation Criteria	Rating Score	Comments
6.	**Overall Performance**<br><br>(Presentation skills, visual aids, shows confidence during Q&A session, etc.)	4	
7	**Suitability of the specialization**<br>(if the study is within the scope of specialization.)	4	
	**Total Score**	27	

Rating Score: 1 = Very Poor, 2 = Poor, 3 = Average, 4 = Good, 5 = Excellent

Decision: **Approved ( )** **Approved with corrections (/)** **Re-write ( )**
Re-presentation ( ) **Rejected ( )**

Signature :

PROF. MADYA DR. HANIS MASTURA YAHYA, PhD (Leeds)
Nutritional Science Program
Centre for Healthy Ageing and Wellness (H-CARE)
Faculty of Health Sciences
Universiti Kebangsaan Malaysia

Name of reviewer : Dr Hanis Mastura Yahya Date : 25/7/2025
